# Distinct genetic architecture underlies the emergence of sleep loss and prey-seeking behavior in the Mexican cavefish

**DOI:** 10.1186/s12915-015-0119-3

**Published:** 2015-02-20

**Authors:** Masato Yoshizawa, Beatriz G Robinson, Erik R Duboué, Pavel Masek, James B Jaggard, Kelly E O’Quin, Richard L Borowsky, William R Jeffery, Alex C Keene

**Affiliations:** Department of Biology, University of Nevada, Reno, Reno, NV 89557 USA; Department of Biology, University of Hawaii, Manoa, Honolulu, HI 96822 USA; Department of Biology, New York University, New York, NY 10012 USA; Department of Biology, Centre College, Danville, KY 40422 USA; Department of Biology, University of Maryland, College Park, MD, 20742 USA; Present address: Carnegie Institution for Science, Department of Embryology, Baltimore, MD 21218 USA

**Keywords:** Sleep, Sensory perception, Astyanax mexicanus, Cavefish, Foraging

## Abstract

**Background:**

Sleep is characterized by extended periods of quiescence and reduced responsiveness to sensory stimuli. Animals ranging from insects to mammals adapt to environments with limited food by suppressing sleep and enhancing their response to food cues, yet little is known about the genetic and evolutionary relationship between these processes. The blind Mexican cavefish, *Astyanax mexicanus* is a powerful model for elucidating the genetic mechanisms underlying behavioral evolution. *A. mexicanus* comprises an extant ancestral-type surface dwelling morph and at least five independently evolved cave populations. Evolutionary convergence on sleep loss and vibration attraction behavior, which is involved in prey seeking, have been documented in cavefish raising the possibility that enhanced sensory responsiveness underlies changes in sleep.

**Results:**

We established a system to study sleep and vibration attraction behavior in adult *A. mexicanus* and used high coverage quantitative trait loci (QTL) mapping to investigate the functional and evolutionary relationship between these traits. Analysis of surface-cave F_2_ hybrid fish and an outbred cave population indicates that independent genetic factors underlie changes in sleep/locomotor activity and vibration attraction behavior. High-coverage QTL mapping with genotyping-by-sequencing technology identify two novel QTL intervals that associate with locomotor activity and include the narcolepsy-associated tp53 regulating kinase. These QTLs represent the first genomic localization of locomotor activity in cavefish and are distinct from two QTLs previously identified as associating with vibration attraction behavior.

**Conclusions:**

Taken together, these results localize genomic regions underlying sleep/locomotor and sensory changes in cavefish populations and provide evidence that sleep loss evolved independently from enhanced sensory responsiveness.

**Electronic supplementary material:**

The online version of this article (doi:10.1186/s12915-015-0119-3) contains supplementary material, which is available to authorized users.

## Background

The ability to adapt, both behaviorally and physiologically, to changing environments is essential for survival [1,2]. One such example is the emergence of adaptive foraging traits in food poor conditions [3]. Diverse species respond to food scarcity by suppressing sleep and enhancing responsiveness to sensory cues, presumably to increase the probability of finding food [4-6]. Sleep is characterized by extended periods of behavioral quiescence that correlate with elevated response thresholds to sensory stimuli [7]. While there is evidence for the gating of sensory stimuli during sleep, little is known about the evolutionary relationship between these processes [8]. The Mexican cavefish, *Astyanax mexicanus* demonstrates evolutionarily-derived sleep loss and enhanced sensory sensitivity presumably to enhance foraging abilities in the nutrient poor environment [9,10]. Here, we examine the genetic and evolutionary relationship between these processes to determine whether evolutionarily derived sleep loss is functionally related to enhanced responsiveness to sensory stimuli.

The Mexican cavefish provides a powerful system for investigating adaptive evolution in a nutrient poor environment. *A. mexicanus* consists of multiple eyed ‘surface’ populations that inhabit rivers in the Sierra de El Abra region of Northeast Mexico and of 29 geographically isolated populations of subterranean dwelling cave-morphs [11-14]. Within the past few million years, at least five independent invasions by two different migration waves of eyed surface fish have established independent cavefish populations [15-18]. Many extant *A. mexicanus* cave populations have independently acquired eye loss and albinism, revealing the convergent evolution of these cave-associated traits [19-22]. Despite their geographic isolation, *A. mexicanus* surface and cave populations are inter-fertile, permitting genetic analysis of the complex, multi-locus traits underlying evolutionary changes [12].

The cave environment is nutrient poor and cavefish populations have evolved robust changes in locomotor and feeding-related traits that include sleep loss and increased non-visual prey seeking, termed vibration attraction behavior (VAB), where cave populations are attracted to vibration at frequencies that prey can produce [9,10]. The enhanced VAB of cavefish is a proxy for sensitivity to sensory stimuli because it is mediated largely by an increase in the number and size of cranial superficial neuromasts (SN) [10,22-25]. Additionally, juveniles from multiple, independently derived cave populations display reduced sleep and elevated locomotor activity that is independent of morphological traits associated with cave populations [9]. While enhanced foraging traits are present in multiple-independently derived populations of *A. mexicanus*, the genetic architecture and physiological processes regulating these behaviors remain unknown.

Here, we investigate the functional and evolutionary relationship between sleep/wake changes and enhanced sensory responsiveness to food in *A. mexicanus*. We identify independently derived sleep loss and enhanced locomotion in multiple adult cave populations, providing the opportunity to examine the relationship between sleep/wake patterns and feeding-associated sensory responsiveness. We used hybrid analysis and high coverage quantitative trait loci (QTL) mapping to examine the genetic architecture underlying the evolution of VAB and enhanced locomotor activity. Analysis of surface-cave hybrids reveals a low correlation between VAB and locomotor activity, indicating that distinct genetic pathways underlie changes in sleep/locomotor behavior and vibration attraction behaviors. These conclusions were reinforced by QTL mapping analysis with genotyping-by-sequencing technology, which revealed distinct genetic loci regulating VAB and locomotor behavior. Therefore, our findings demonstrate the independent co-evolution of sleep and enhanced sensory responsiveness.

## Results and discussion

**Figure 1:**
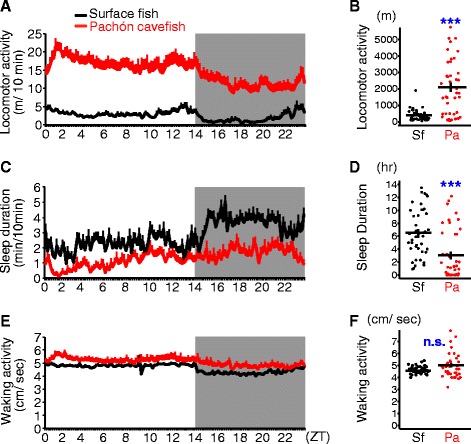
**Elevated locomotor activity and sleep loss in adult** ***A. mexicanus*** **cavefish** ***.*** **(A)** Total swimming distance (meters) is significantly greater in Pachón cavefish (red line) during the day (unshaded; ZT 0 to 14) and night (shaded; ZT 14 to 24) than surface fish (black line). Bars at each 10 minutes represent mean + standard error of mean (s.e.m.). **(B)** Quantification of total 24 hour activity reveals significantly enhanced locomotor activity in Pachón cavefish (U = 373, *P* <0.001). Each dot represents the individual phenotypic value. Black horizontal and vertical lines indicate mean ± s.e.m. **(C, D)** Sleep profile and total 24 hour sleep reveal reduced sleep in Pachón cavefish (red) compared to surface fish (black). (t_83_ = 4.3, *P* <0.001). **(E, F)** Averaged waking activity in each 10 minutes does not differ between surface fish (black) and Pachón cavefish (red) (U = 767, *P* = 0.232). N = 42, 43 for surface fish and Pachón cavefish, respectively, for all assays. Sf: surface fish. Pa: Pachón cavefish. *** denotes *P* <0.001. n.s.: not significant.

To determine the relationship between sleep traits and VAB, we developed an assay for tracking the activity of adult *A. mexicanus*. The locomotor activity of individually housed adult fish was measured over 24 hours and total distance traveled was determined. Consistent with data for juvenile cavefish [9], total locomotor activity of Pachón cave populations was significantly greater than surface fish at all time points tested, although adult individuals showed more variation than juveniles (Figure 1A, B; [9]). Interestingly, despite the dramatic increase in activity, we observed diurnal rhythms under light:dark conditions in both populations of fish (Figure 1A). Therefore, while the overall activity of Pachón cavefish is increased compared to surface fish, they retain diurnal rhythms in the absence of functional eyes.

The elevated activity of Pachón cavefish could be due to hyperactivity or reduced sleep. Convergent evolution of sleep loss has previously been reported in juvenile fish at 21 to 27 days post fertilization, but it is not known whether sleep and activity differ in adult cavefish [9]. Across phyla, sleep is characterized by extended periods of quiescence, defined in larvae and juveniles as immobility of >60 seconds in *A. mexicanus* and zebrafish, and elevated response threshold [9,26,27]. We established a sleep assay in adult *A. mexicanus* by assaying the response thresholds to determine the minimum movement that constitutes behavioral quiescence (see Additional file 1A-D). Electric shock was used as the stimulus to measure the response threshold because sensitivity to light and mechanical stimuli differs between cave and surface populations [28]. Surface and cavefish were provided increasing levels of electric shock and responsiveness was measured by comparing movement prior to and following the stimulus. Surface fish that moved less than 4 cm/second for the minute prior to stimulation showed the lowest probability of response to 10 mA electrical shock (see Additional file 1B,C), and, therefore, this parameter setting was used to define behavioral quiescence associated with sleep. In addition, we also confirmed that the immobility of >60 seconds increased the response threshold in our system (see Additional file 1D). Analysis of sleep duration revealed Pachón cavefish sleep significantly less than surface fish confirming that sleep loss previously observed in larvae is conserved in adults (t_83_ = 4.3, *P* <0.001; Figure 1C, D). Quantification of light–dark sleep differences reveal both surface fish and cavefish sleep more during the night, suggesting diurnal rhythms in the absence of visual capacity in Pachón cavefish (Additional file 2B). Waking activity, defined as the average velocity in every 10 minutes when the animal is not asleep, does not differ between surface and cavefish indicating that the enhanced locomotor activity in the Pachón cave population is not due to hyperactivity (U = 767, *P* = 0.232, N = 42, 43 for surface fish and cavefish, respectively. Figure 1E,F, and Additional file 2C).

Sleep loss can occur through a reduction in the number of sleep bouts, shortened sleep bout duration, or a combination of both components. We found the sleep bouts length is shortened and average bout number is reduced in adult Pachón cavefish compared to surface fish indicating that both initiation and maintenance of sleep are altered in Pachón cavefish (repeated-measures two-way analysis of variance (ANOVA): F_1,83_ = 16.3, *P* <0.001, and F_1,83_ = 31.0, *P* <0.001, respectively; Additional file 2D-G). Bout duration was lengthened during the night-phase in both surface and Pachón fish, raising the possibility of functional differences between day and night immobility (repeated-measures two-way ANOVA: F_1,83_ = 30.1, *P* <0.001; Additional file 2D, F. See figure legends, too.). Taken together, these results indicate that sleep is reduced in adult cavefish providing the opportunity to examine interactions between sleep and foraging behavior related to enhanced responsiveness to sensory stimuli.

**Figure 2:**
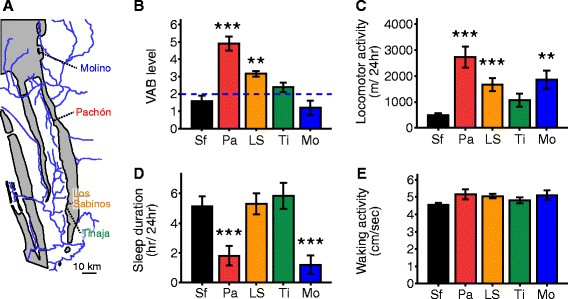
**Independent evolution of foraging behavior and sleep-related traits in adult** ***A. mexicanus.*** **(A)** Geographic location of caves in the Sierra del El Abra region of Northeast Mexico. Gray shadings indicate limestone mountain ranges, and blue lines indicate primary river systems. **(B)** The Pachón (Pa), Los Sabinos (LS) populations of cavefish display greater vibration attraction behavior (VAB) than surface fish (Sf), Tinaja (Ti) and Molino (Mo) populations. (Kruskal-Wallis χ^2^ = 43.1, df = 4, *P* <0.001; *post hoc* test with Bonferroni adjustment comparing surface fish with: Pachón, *P* <0.001; Los Sabinos, *P* <0.01; Tinaja, *P* >0.05). N = 19, 19, 20, 20 and 10 for surface fish, and Pachón, Los Sabinos, Tinaja and Molino cavefish, respectively. **(C)** Locomotor activity over the 24 hour test period was significantly greater in Pachón, Los Sabinos, and Molino compared to surface fish. Activity was not enhanced in the Tinaja population (non-parametric Kruskal-Wallis test: χ^2^ = 28.9, df = 4, *P* <0.001; *post hoc* test with Bonferroni correction comparing surface fish with: Pachón, *P* <0.001; Los Sabinos, *P* <0.001; and Molino, *P* <0.01). N = 23, 23, 19, 19 and 11 for surface fish, Pachón, Los Sabinos, Tinaja and Molino cavefish, respectively. **(D)** Sleep duration in 24 hours was significantly reduced in Pachón and Molino populations compared to surface fish (one-way ANOVA, F_4,90_ = 7.9, *P* <0.001; *post-hoc* Dunnett t test was applied between surface fish and each cavefish population). No difference was observed in the Los Sabinos and Tinaja populations. **(E)** Waking activity (cm/second) did not differ between any of the four cavefish populations and surface fish. (Kruskal-Wallis test: χ^2^ = 5.1, df = 4, *P* = 0.277). *** denotes *P* <0.001, ** denotes *P* <0.01.

We measured sleep and activity in three additional cavefish populations to determine whether evolutionary changes in sleep were present in multiple cave populations, and their relationship to VAB (Figure 2 and Additional file 3). The Molino cave population is derived from the more recent migration wave of ancestral surface fish populations [18,29]. We also tested Tinaja and Los Sabinos cave populations that are derived from a different ancestral surface fish population that is distinct from the Pachón phylogenetic group [16,18,30,31]. We assayed VAB by measuring the number of approaches to a 35 Hz vibrating glass rod over a three minute assay [10]. In agreement with previously reported results, the Pachón and Los Sabinos populations display VAB, while this is absent in Molino and surface fish [10]. Additionally, we did not detect any significant difference in VAB between Tinaja cavefish and surface fish revealing highly variable levels of VAB between different cave populations (Figure 2B).

The total locomotor activity was greater in Pachón, Los Sabinos and Molino cave populations compared to surface fish revealing the evolutionary convergence of enhanced locomotor behavior (Figure 2C). There were no significant differences in locomotor activity between surface fish and Tinaja cavefish (Figure 2C; Mann–Whitney U = 147, *P* = 0.284 with Bonferroni correction). The enhanced locomotor activity of Molino cave populations is due to a reduction in sleep duration with no difference in waking activity compared to surface fish (one-way ANOVA: F_4,90_ = 7.9, *P* <*0*.001, and Kruskal-Wallis χ^2^ = 5.1, df = 4, *P* = 0.277, respectively; Figure 2D, E). The Los Sabinos cave population did not display changes in sleep or waking activity, an indicator of hyperactivity, compared to surface fish, suggesting that the enhanced total locomotor activity may be due to contributions of both these factors that are individually below detectable significance (Figure 2D, E). Because Molino and Pachón cavefish were derived from different ancestral lineages, these findings reveal a convergence on evolutionarily derived sleep loss. Total sleep or locomotor activity in Tinaja cavefish did not differ from surface fish, raising the possibility of cave-specific selection pressure that resulted in sleep-activity differences between cave populations. Further, with the exception of Pachón, no relationship was observed between the presence of VAB and sleep, suggesting these traits are under independent functional and selective regulation. Taken together, these findings identify reduced sleep in the independently derived Pachón and Molino cave populations, revealing the convergent evolution of sleep loss in adult *A. mexicanus* cavefish.

**Figure 3:**
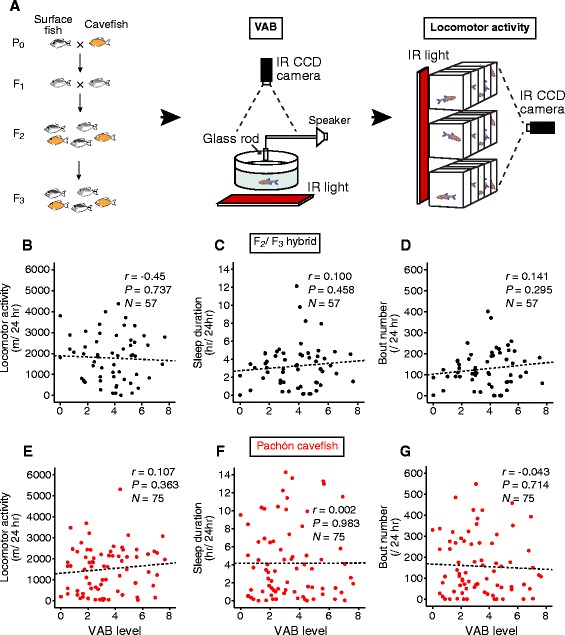
**VAB does not segregate with sleep or locomotor activity in hybrid or outbred cavefish populations. (A)** F_2_ and F_3_ hybrid fish were generated from a female surface fish and a male Pachón cavefish. Hybrid fish were then tested for vibration attraction behavior (VAB) followed by locomotor activity **(B-D)**. No correlation was observed between VAB and locomotor activity **(B)**, sleep duration **(C)** and bout number **(D)** (*P* >0.05). **(E-G)** Pachón cavefish were assayed for VAB and locomotor behavior. No correlation was observed between VAB and locomotor activity **(E)**, sleep duration **(F)** and bout number **(G)** (*P* >0.05).

Enhanced locomotor activity and VAB are proposed to be adaptive traits that improve the probability of finding food in a nutrient-poor environment [9,10,32]. It is possible that conserved genetic architecture underlies VAB and sleep loss in Pachón cavefish. Alternatively, these traits may have distinct genetic mechanisms that underlie the co-evolution of these traits in Pachón cavefish. To differentiate between these two possibilities, we generated F_2_ and F_3_ hybrid fish from a cross of surface fish and Pachón cavefish and individual fish were assayed for sleep duration, locomotor activity and VAB (Figure 3A). All three traits were highly variable in hybrid fish allowing for analysis of trait segregation. The presence of VAB did not correlate with locomotor activity, sleep duration or bout number in surface-Pachón hybrids, suggesting that distinct genetic mechanisms underlie the regulation of locomotor activity and VAB (Figure 3B-D).

Trait analysis within a single population provides further insight into the naturally occurring genetic variation that regulates behavior. We found highly variable sleep and VAB in an outbred population of Pachón cavefish that is likely due to standing genetic variation. Individual outbred Pachón cavefish were assayed for sleep-associated behaviors and VAB to further examine the relationship between these traits. No correlation was detected between VAB and locomotor activity, sleep or sleep bout number fortifying the conclusion that these traits are independently regulated (Figure 3E-G). Therefore, analysis of both surface-Pachón hybrid fish and naturally occurring genetic variation in an outbred Pachón population indicated that VAB and locomotor activity are regulated by distinct genetic mechanisms.

**Figure 4:**
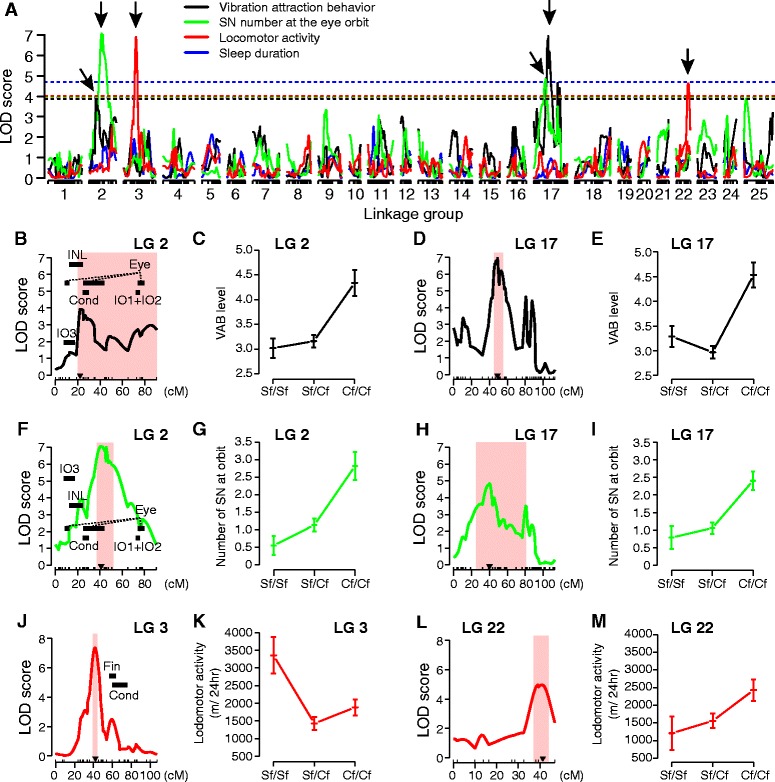
**QTL reveals distinct genetic architectures controlling VAB and sleep. (A)** Logarithm of the odds (LOD) scores computed with single-QTL model genome-scan are plotted against the distance across each linkage group (LG). Black solid lines indicate LOD scores for VAB-level, green lines for SN number at the eye orbit, red lines for locomotor activity, and blue lines for sleep duration. Significant QTL for VAB and SN number at the orbit were detected at LG 2 and 17. Significant locomotor activity-QTL was detected at LG 3 and 22 following multiple QTL mapping. The horizontal dotted lines indicate the genome-wide significance thresholds at *P* <0.05. Arrows indicate the peaks of the significant QTL. **(B-E)** LOD scores for VAB at LG 2 **(B)** and LG 17 **(D)**. The X-axis indicates genetic distance in centimorgans (cM), and red colored shades denote Bayesian credible intervals with probability coverage as 0.95 for each significant QTL. Effect plots of phenotypic values of VAB against each genotype (mean ± s.e.m.) at the peak locus denoted by the black triangle in **(B)** and **(D)** are shown in **(C)** and **(E)**, respectively. **(F-I)** LOD scores for the number of superficial neuromasts (SN) at LG 2 **(F)** and LG 17 **(H)**, and effect plots of phenotypic values of SN number against each genotype at LG 2 **(G)** and at LG 17 **(I)**. **(J-M)** LOD scores for locomotor activity at LG 3 **(J)** and LG 22 **(L)**, and effect plots of phenotypic values of SN number against each genotype at LG 3 **(K)** and at LG 22 **(M)**. Black bars in **(B)**, **(F)** and **(J)** indicate the reported QTL intervals for relative condition (Cond), eye size (Eye), fin placement (Fin), SO3 width (IO3) [34], thickness of inner nuclear layer (INL, [33]), SO1 and SO2 fusion (IO1 + IO2, [35]. Sf/Sf, surface fish homozygote, Sf/Cf, heterozygote, and Cf/Cf, cavefish homozygote.

Previous modeling studies examining sleep loss in juvenile cavefish suggested a small number of genomic loci underlie changes in sleep, but this has not been directly examined [9]. We, therefore, sought to map genomic regions associated with sleep and activity changes in cavefish by identifying quantitative trait loci that associate with locomotor regulation or sleep. F_2_ and F_3_ surface-cave hybrids were behaviorally phenotyped and genotyped with 698 markers of genotyping-by-sequencing (GBS), microsatellite and candidate gene-single nucleotide polymorphisms (SNP) to localize genomic regions regulating VAB and locomotor activity [33]. This approach yields more precise positions for QTL mapping than has previously been reported in mapping of behavioral traits including VAB [24]. Consistent with our previously published QTL map, this new approach identified QTL on linkage groups (LG) 2 and 17, which are congruent with QTL for VAB level and SN on the eye orbit (Figure 4A) [24].

While the QTL intervals identified regulating VAB and activity are large, we sought to find syntenic regions in the zebrafish genome and identify candidate genes that may underlie the given phenotypes [36]. We mapped our candidate QTL intervals, defined by 95% Bayesian credible intervals, to the zebrafish genome and identified syntenic regions on zebrafish chromosomes 5, 20 and 24 for LG 2 and chromosome 8 for LG 17 (see Additional file 4). These regions contain a number of candidate genes controlling VAB level including *proprotein convertase subtilisin/kexin type 5a*, which is involved in the development of mechanosensory neuromasts (Figure 4B-I, Additional file 4) [37]. In addition, two QTL were detected for the locomotor activity at LG 3 and 22 (Figure 4A, J-M). Despite the functional relationship between sleep and locomotor activity we did not identify any significant QTL regulating sleep, perhaps due to increased variability associated with sleep measurements, or distinct mechanisms regulating locomotor activity and sleep. To detect the QTL with the effect size more than 17% as we observed, the current QTL mapping experiment requires a sample size greater than 125 (estimated by qtlDesign software [38,39]). In these experiments, the number of animals used is limited due to long-term behavior assays and the use of adult fish. While the small number of individuals used in locomotor activity analysis limits statistical power (N = 58) (Additional file 4), the approximate position of QTL would likely remain the same. Therefore, the most significant QTL demonstrate distinct loci regulating locomotor activity and VAB despite the relatively low N value of the animals used.

The Pachón cavefish alleles at LG 3 reduce locomotor activity, while alleles at LG 22 enhance, indicating that the QTL at LG 22 is more relevant for the enhancement of activity observed in Pachón cavefish (Figure 4J-M, Additional file 4). A number of genes located within the LG 22 syntenic regions of the zebrafish genome (chromosome 7 and 8; Additional file 4) that are potential regulators of sleep/locomotor activity include the narcolepsy-associated *tp53 inducible protein*, *dopamine receptor D4b*, and the *nicotinic cholinergic receptor*, *alpha 7*. These findings provide more precise genomic loci regulating VAB and define two novel loci regulating locomotor behavior. Further, we introduce candidate genetic regulators of activity and enhanced vibration response that may be examined further in another model organism, zebrafish [22], or using targeted gene disruption in cavefish. As an initial step in validating *dopamine receptor D4b* as a candidate regulator of locomotor activity, we examined the effects of the dopamine receptor *D2, D3,* and *D4* antagonist haloperidol on locomotor activity in *A. mexicanus.* Treating fish with haloperidol significantly reduced locomotor activity in Pachón cavefish and trends towards significance in surface fish (see Additional file 5), indicating that dopamine signaling inhibits sleep in *A. mexicanus* as has previously been reported in vertebrate and invertebrate systems [40,41]. While selective *D4b* or *D4* antagonists are not available in quantities practical for testing in adult fish, these findings provide an initial step towards validating genes identified through QTL mapping. Future studies that utilize a recently sequenced genome in Pachón cavefish may allow for genome-wide association studies of an outbred cavefish that will provide increased resolution of candidate genes underlying VAB and sleep. Importantly, we identify distinct QTL that regulate sleep and VAB, fortifying the behavioral analysis in hybrid animals indicating that independent genetic architecture regulates sleep and VAB.

Our findings demonstrate the independent evolution of two foraging-related traits in the Mexican cavefish. We establish a paradigm for measuring sleep in adult *A. mexicanus* and demonstrate that the sleep loss previously observed in juvenile cavefish is also present in adult fish. We observed reduced sleep in two populations of cavefish from independent lineages and increased activity in three cave populations compared to ancestral-type surface fish. The Molino and Pachón populations are derived from separate ancestral stocks that are also phylogenetically distinct from the origin of the Tinaja and Los Sabinos populations [18]. The presence of sleep loss in Molino and Pachón fish demonstrates the convergent evolution of sleep loss in these cave populations. In addition, we find significantly enhanced VAB in cavefish from the Pachón and Los Sabinos populations, but not from the Molino and Tinaja caves. Therefore, both VAB and sleep/locomotor changes are variable between cave populations, and these two behavioral traits are likely independently derived.

Morphologically, Los Sabinos and Tinaja populations are similar to Pachón and Molino. It is, therefore, of particular interest that there are robust behavioral differences between these populations. Behavioral traits appear to be more flexible than morphological traits across cave populations, possibly to facilitate the adaptation to each ecological demand [42-47]. This notion is supported by the example of Tinaja and Los Sabinos cavefish, which are in close geographic proximity and share a close phylogenetic history [15,30]. While sleep duration does not differ between these two populations, they are significantly different in VAB level and locomotor activity. The ecosystems of these populations are similar but food sources may vary in each location, raising the possibility that differences in food availability between caves underlie differences in foraging traits [14] (M Yoshizawa, personal observation). The Los Sabinos cave is inhabited by a large bat colony where cavefish swim to and forage on bat droppings, whereas no obvious bat colony exists above the Tinaja cave pool (M Yoshizawa, personal). We also observed thick soil covering the Tinaja cave floor and at the bottom of the cave pool suggesting that Tinaja cavefish may primarily forage on organic matter in the substrate rather than bat guano. Thus, the presence of moving food including bat droppings may contribute to Los Sabinos cavefish evolving active foraging strategies seen in VAB and increased locomotor activity that are not observed in Tinaja cavefish.

Robust sleep loss was previously reported in juvenile cavefish at 21 to 27 days post fertilization from Pachón, Molino and Tinaja populations [9]. It is of particular interest that sleep loss was not observed in adult Tinaja cave populations, raising the possibility that distinct genetic mechanisms govern sleep loss in juvenile and adult fish. It has been proposed that sleep suppression in juvenile cavefish is due to altered noradrenergic signaling because the beta-blocker propranolol restores surface-fish levels of sleep in Pachón populations [32]. We have found that propranolol has no effect on the sleep of adult Pachón fish, fortifying the notion that distinct mechanisms regulate sleep across developmental stages (see Additional file 2H, I). The finding that sleep duration is dramatically reduced in Tinaja juveniles, but not adults raises the possibility that distinct evolutionary pressures underlie sleep loss in juvenile and adult cavefish. For example, juveniles may need to feed continuously to grow quickly and compete with their adult conspecifics. In addition, there is a possibility that juvenile and adult fish have different diets, possibly resulting in cave-specific differences in sleep and foraging. A more detailed investigation of cave ecology and animal diets may provide insight into the developmental differences in sleep in Tinaja cavefish.

Distinct genomic architecture regulating locomotor activity and VAB in Pachón cavefish presents a mechanism to facilitate the colonization in different caves. Since each cave has minor differences as described above, cavefish ancestors may modulate sensory-based behavior and locomotor behavior differently [48,49]. It is interesting that the standing variation of locomotor activity in surface fish is very small (Figure 1A) while the one of VAB was large [23]. It is possible that enhanced locomotor activity evolved through *de novo* mutation whereas VAB is thought to be derived from the selection of the standing variation in surface population. The available Pachón cavefish genome is an excellent resource to start surveying the selection in these QTL regions [50]. Comparative genomics between surface fish and Pachón, Tinaja, Los Sabinos or Molino in these QTL intervals may reveal the different microevolutionary selection regions among these cave species [51]. This will further provide an insight on the evolutionary strategy that this vertebrate takes to adapt to, and survive in, an extreme condition.

Recently, cavefish were shown to have a mutation in monoamine oxidase that results in changes in the levels of numerous neurotransmitters, raising the possibility that a simple genomic change alters diverse behaviors [52]. The identification of sleep loss in adult cavefish provides the opportunity to examine the relationship between sleep loss and other foraging traits previously identified in adult cavefish. Here, we examined the relationship between sleep and VAB. Three lines of evidence indicate independent and convergent evolution for these foraging related traits. First, Molino cavefish do not display enhanced VAB, but have a dramatic reduction in sleep compared to surface fish (Figure 2B,C). Second, there was no detectable correlation between VAB and sleep duration/locomotor activity in surface-Pachón hybrid or outbred Pachón populations, supporting the notion that an independent genetic factor(s) regulates each trait. Finally, different QTL were identified as regulating each trait, supporting the notion that distinct genetic architecture underlies these foraging-related traits. Therefore, these findings reveal the independent evolution of foraging behaviors, sleep and VAB, in the Mexican cavefish. It is likely that these traits evolved as genetically distinct components to foraging behavior. The evolution of individual subcomponents of complex behavior has previously been described for tunnel burrowing behavior in beach mice, raising the possibility that modularity of behavioral subcomponents enhances behavioral adaptability [53].

Diurnal activity patterns were detected in each of the cave populations tested for sleep. These cave populations lack functional eyes, and, therefore, it is not clear how light input reaches the brain. Cavefish possess a functional pineal gland that is required for behavioral responses to light in a shadow-response assay, and, therefore, it is possible that melatonin release from the pineal regulates light–dark differences in activity and sleep [54,55]. All fish were tested under light–dark conditions and, therefore, it is not clear whether the diurnal activity patterns are due to circadian rhythms or a masking effect of light due to the presence of light. Light entrainable-circadian rhythms are absent in the blind Somalian cavefish *Phreatichthy andruzzii,* but these fish maintain food-entrainable rhythms [56]. The genome of *A. mexicanus* Pachón cavefish retains light-inducible circadian genes, but their molecular clocks are not entrainable, at least partially due to constitutively high expression of the circadian gene, *period2* [57]. Future work examining sleep and activity patterns under conditions of constant darkness will reveal whether the observed diurnality results from a masking effect of light or a functional circadian clock.

## Conclusions

The Mexican cavefish *Astyanax mexicanus* is a powerful model to study the relationship between foraging behaviors. We demonstrate that evolutionarily derived sleep loss is conserved in adult cavefish and distinct evolutionary paths and genetic mechanisms underlie sleep loss and enhanced sensory responsiveness. High coverage QTL mapping revealed the first loci underlying locomotor activity that are distinct from those regulating any previously identified behaviors. These findings provide a more complete picture of the functional and evolutionary relationship between sleep and sensory responsiveness and further our understanding of the emergence of sleep in response to ecological changes.

## Methods

### Fish maintenance and rearing

Fish were housed in the University of Nevada-Reno core facility with 21°C ± 0.5°C water temperature for rearing and behavior experiments and 23°C ± 0.5°C water temperature for breeding. Lights were maintained on a 14:10 light/dark cycle throughout the animal’s lifetime [58-60] for both rearing and behavior experiments with a light intensity of approximately 25 Lux. Fish husbandry was performed as previously described [24,58,59]. Fish were raised to adults and maintained in standard 42 L tanks in a commercial tank system (Marineland Integrated Rack System MV8FRT, Blacksburg, VA, USA). Adult fish were fed a mixture diet of black worms to satiation twice daily at ZT2 and ZT12 (California Blackworm Co., Fresno, CA, USA) and standard fish food during periods when fish were not being used for behavior experiments or breeding (Tetramine Pro, Tetra, Blacksburg, VA, USA). In order to individually identify animals used in QTL analysis, fish were isolated in a 76 L filtered tank containing a custom net housing composed of 25 chambers (each chamber is 9 × 6 × 7 cm). All fish tested for behavioral experiments were between 3 and 5 cm in standard length. Hybrid fish in QTL experiments were 5- to 7-years old while all other fish used were 1 to 3 years of age. No relationship between age and sleep duration, locomotion activity or VAB was observed (*r* = −0.172, 0.116 or 0.052, respectively; both *P* >0.10, N = 81 Pachón cavefish) and, therefore, age was not factored into QTL analyses.

### Sleep and locomotor behavior

Fish were recorded under standard conditions in a custom-designed 8.7 L recording chamber with opaque partitions that allow for five individually housed fish per tank. The recording chamber was illuminated with a custom designed IR LED source (Infrared 850 nm 5050 LED Strip Light, Environmental Lights, San Diego, CA, USA). Behavior was recorded for 24 hours beginning one hour after lights on (ZT1) after 4 to 5 days acclimation in recording chambers. Videos were recorded at 15 frames/second using a USB webcam (LifeCam Studio 1080p HD Webcam, Microsoft, Redmond, WA, USA) fitted with a zoom lens (Zoom 7000, Navitar, Rochester, NY, USA). An IR high-pass filter (Optical cast plastic IR long-pass filter, Edmund Optics Worldwide, Barrington, NJ, USA) was placed between the camera and the lens to block visible light. Videos were captured by a video capturing software, (Version 1.10.4), and were subsequently processed using Ethovision XT 7.1 (Noldus, IT, Wageningen, Netherlands). Water temperature was monitored throughout the recordings and no detectable differences were observed during the light and dark periods. The visible light during behavior recordings was approximately 25 Lux. Tracking parameters for detection were set as follows: detection set to subject brighter than background and brightness contrast from 20 to 255; current frame weight set to 15; video sample rate set to 15 frames/seconds, and pixel smoothing turned off. Data were subsequently processed using custom-written Perl scripts (v5.10.0) and Excel macro (Microsoft). To define sleep in *A. mexicanus*, we first observed the fish state while they were inactive. *A. mexicanus* surface fish stayed at the same spot or slowly drifted by occasionally making light beats with their tail fin to stabilize their horizontal posture. Based on the actogram, we defined that 4 cm/second is the threshold to distinguish locomotor and passive drift activity (comparing actogram as in Additional file 1B with fish movements in the recorded videos). To measure the response threshold, we applied electrical stimuli in the range of 10 to 80 mA in the water conductivity as 600 to 800 μS/cm. The recording chambers were fitted with stainless steel-mesh sidewalls (see Additional file 1A). Stimuli were generated with Powerpac 1000 (Bio-Rad Laboratories Inc., Hercules, CA, USA) and the timing was controlled manually (approximately 0.5 second) and applied once per experiment. We surveyed the association between the duration of inactivity (10 sec to 2 minutes) and the response to stimulus (>4 cm/second), and found that 1 minute- or 1.5 minute-inactivity corresponds to the increase of the threshold of the response at 10 mA stimulus (lower limit of this device, Additional file 1B, C). Fish always responded to the 60 mA and higher stimulus regardless of the duration of inactivity (Additional file 1B, C). In addition, immobility of more than one minute increased the response threshold (Additional file 1D). Therefore, for all experiments we concluded that one minute of inactivity corresponds to an elevated response threshold of *A. mexicanus* and inactivity bouts of >1 minute was used to define sleep. A total of 10 surface fish were used for this experiment. Locomotor activity was measured as the sum swimming distances in every 10 minutes (Figure 1) or 24 hours while fish were awake. Sleep durations were measured as the sum of immobility duration in every 10 minutes (Figure 1) or 24 hours while fish slept. Bout durations were averaged measurements of the sleep duration per bout in every 10 minutes, and bout numbers are the numbers of sleep bouts per 10 minutes or per hour (see Additional files 2 and 3). Waking activities were averaged swimming velocity per 10 minutes while fish were awake. Note, waking activity in every 10 minutes masks detailed fish motions, including thrusting and stopping so that the locomotor activity is not simply the result of (waking duration) × (waking activity) in our setup.

### Vibration attraction behavior

We assayed VAB as described previously [10,23,24]. Individuals were briefly acclimated in a cylindrical assay chamber (Pyrex 325 ml glass dish, 10 cm diameter 5 cm high, Corning, Corning, NY, USA) filled with conditioned water (pH 6.8; conductivity approximately 600 μS) for four to five days prior to the assay. During testing, vibration stimuli were generated with a 7.5 mm-diameter glass rod vibrating at 35 Hz using a Leader LG1301 function generator (Leader Instruments Corp., Cypress, CA, USA) with an audio speaker (Pro Speakers, Apple, Cupertino, CA, USA). The number of approaches (NOA) to the vibrating rod was video recorded during a three-minute period under infrared illumination (880 nm wave length, BL41192-880 black light, Advanced Illumination, Rochester, VT, USA), and counted using ImageJ 1.42q software (National Institutes of Health, Bethesda, MD, USA).

### Genotyping-by-Sequence QTL analysis

We previously isolated 463 SNP markers by employing GBS (or sequenced restriction-site associated DNA tags: RAD-seq) and 235 microsatellite and SNP (TaqMan method) markers covering most of the *A. mexicanus* genome [33]. Detailed methods for linkage group construction and marker selection were described in [33]. Our method for RAD-seq library construction and sequencing followed previously described protocols [61], with a few exceptions. Briefly, we extracted genomic DNA from 115 F_2_ hybrid individuals using a DNeasy Blood and Tissue extraction kit (Qiagen, Valencia, CA, USA). After quantification of samples, each 1 μg of genomic DNA was digested with the restriction enzyme *SbfI*-HF (New England Biolabs, Ipswich, MA, USA). We ligated one of 32 unique Illumina Solexa© P1 adaptors to each sample and then combined the DNA from 32 individuals into a common library. We randomly sheared the DNA in each library using an ultrasonicator and then size-selected fragments between 300 to 500 bp. After cleaning the fragments, we performed blunt-end repair and added adenine overhangs to the 3′ ends of each DNA library, and then, ligated a single Illumina Solexa© P2 adaptor to each DNA library. We performed fragment cleaning in each step above then amplified this library for 16 cycles in PCR using modified amplification primers and Phusion Taq DNA polymerase (New England Biolabs, Ipswich, MA, USA). Following quantification of each amplified library using an Agilent Bioanalyzer (Santa Clara, CA, USA), we sequenced them in single-end for 100 cycles on an Illumina HiSeq 1500 at the University of Maryland Institute for Bioscience and Biotechnology Research. Following sequencing, we filtered the raw sequence reads for quality (Q20 across 90% of the read) using the FASTX toolkit. We used the program Stacks v0.998 [62] to perform the remainder of our genotyping analysis. Within Stacks, we used the function *process_radtags* to further filter reads that did not contain complete *SbfI* and barcode sites, and to sort individuals within each library based on the sequence of their 5 bp barcode. We then used the function *denovo_map.pl* to: (1) assemble the reads of each individual into unique loci (called ‘stacks’) and identify heterozygous alleles; (2) match orthologous stacks from the surface fish and cavefish P_0_ into a common catalog of loci and identify SNPs between them; and (3) match the stacks of all F_2_ against this parental catalog and infer genotypes at all loci.

Following sequence assembly and genotyping, we filtered the final dataset by excluding markers that: (1) were not differentially fixed between the surface fish and cavefish P_0_; (2) included missing genotypes in more than 25% of the F_2_; (3) had genotype frequencies that were outside of Hardy-Weinberg equilibrium at a Bonferroni-corrected threshold of α = 0.05; and (4) exhibited a maximum pair-wise recombination fraction greater than 90.9%. This latter criterion is used to exclude alleles which may be ‘switched’ (for example, surface fish alleles called as cavefish alleles), indicating possible genotyping error in the P_0_ [38]. Finally, in addition to these RAD-seq loci, we also included 235 microsatellite and candidate gene markers that were identified in previous studies [19,24,36]. Followed by these genotyping analyses, we performed genetic linkage and QTL analyses in the program R/qtl [63] following the protocols described in Broman and Sen [38]. For genetic linkage mapping, we grouped the RAD-seq and microsatellite markers into linkage groups by specifying a maximum recombination distance of 0.35 and minimum LOD threshold of 6. We then ordered markers along each linkage group by iteratively rippling the order of eight markers at a time and choosing the order that required the fewest crossovers. We performed larger-scale changes in marker order manually and then estimated genetic distances using the Kosambi [64] map function. Finally, we calculated a LOD score for each genotype in order to identify statistically unlikely events, such as double crossovers within a small region of the genetic map. We excluded these potentially erroneous genotypes from further analysis. After above analyses, this linkage map consisted of 698 markers, 1,835.9 cM in total length with the median marker spacing as 1.2 cM and 28.0 markers per linkage group on average. Following linkage mapping, we scanned the genome for QTL associated with the sleep phenotypes using *stepwiseqtl*, a model selection algorithm for multiple QTL mapping [38,65]. We calculated the LOD of association between the genotypes at each marker (markers were simulated every 1.0 cM) and sleep-related phenotypes (the sleep duration, swimming distance, bout number, bout duration or waking activity) and using Haley-Knott regression, while including age at sampling (5- to 7-years old) as covariates. We assessed the statistical significance of the resulting LOD scores by calculating the 95th percentile of genome-wide maximum penalized LOD scores for each retinal layer using 1,200 random permutations of the genotypic and phenotypic data. We defined confidence intervals for the position of the final QTL using 95% Bayesian credible intervals expanded to the nearest genotyped marker.

### Survey of candidate genes in QTL credible intervals

To anchor the *A. mexicanus* genomic intervals on the zebrafish genome (Zv9), we first downloaded the latest version of the *A. mexicanus* genome (AstMex102) from the Ensembl genome browser. We then built a searchable database of this genome and BLASTed the consensus sequences of each GBS-seq and microsatellite locus to this database using the ‘blastn’ option of the program blast v2.2.25+ [66]. After we built the scaffold list of the top hit, we searched annotated genes in AstMex102 gene build (release at Dec 2013), and pulled out these gene information from the zebrafish gene list downloaded from BioMart at Ensembl with attribute for Ensembl Gene ID, Ensembl Transcript ID, Phenotype description, Description, ZFIN symbol, Chromosome Name, Gene Start (bp) [67]. Within the syntenic interval in the zebrafish genome, we searched for candidate genes implicated in the function for foraging, locomotion, sleep or the development or function of sensation and/or superficial neuromast, by narrowing down the candidate genes with BioMart filter, PHENOTYPE.

### Statistics

Parametric tests, including student’s t-test or one-way ANOVA test, were applied if Levene’s equality of variance test was not significant whereas non-parametric Mann–Whitney or Kruskal-Wallis test were applied in the other cases (Figures 1 and 2). *Post hoc* Dunnett t test or Bonferroni correction was applied to compare each population value with the surface fish value using a parametric or non-parametirc method, respectively (Figure 2). Pearson’s correlation analysis was applied for correlation analyses in Figure 3. The above calculations were conducted using IBM SPSS 22.0.0 software (IBM, Somers, NY, USA).

### Ethics statement

All research was approved by the University of Nevada, Reno IACUC Committee under Protocol 00535.

## Additional files

Additional file 1:
**Parameter selection for sleep-like state in** ***A. mexicanus.***
Additional file 2:
**Diurnal activity patterns in surface fish and Pachón cavefish.**
Additional file 3:
**Detailed analysis of sleep and activity related traits in multiple cavefish populations.**
Additional file 4:
**A summary of the location and effect size of significant QTLs for foraging- and sleep-related traits.**
Additional file 5:
**The dopamine receptor antagonist haloperidol modulates sleep and locomotor activity in A. mexicanus.**

## Abbreviations

ANOVA: analysis of variance
bp: base pair
Cf: cavefish
GBS: genotyping by sequencing
IO3: third infraorbital bone
LG: linkage group
LOD: logarithm of the odds
NOA: numbr of approaches
QTL: quantitative trait loci
Sf: surface fish
SN: superficial neuromast
SNP: single nucleotide polymorphism
SO3: third suborbital bone 3
VAB: vibration attraction behavior
