# Supplementary material for: Distinct genetic architecture underlies the emergence of sleep loss and prey-seeking behavior in the Mexican cavefish
Source: BMC Biol. 2015 Feb 20;13:15. doi: 10.1186/s12915-015-0119-3 (PMC4364459; doi:10.1186/s12915-015-0119-3)
Supplement: Additional file 1: — Parameter selection for sleep-like state in A. mexicanus. [file 12915_2015_119_MOESM1_ESM.pdf]

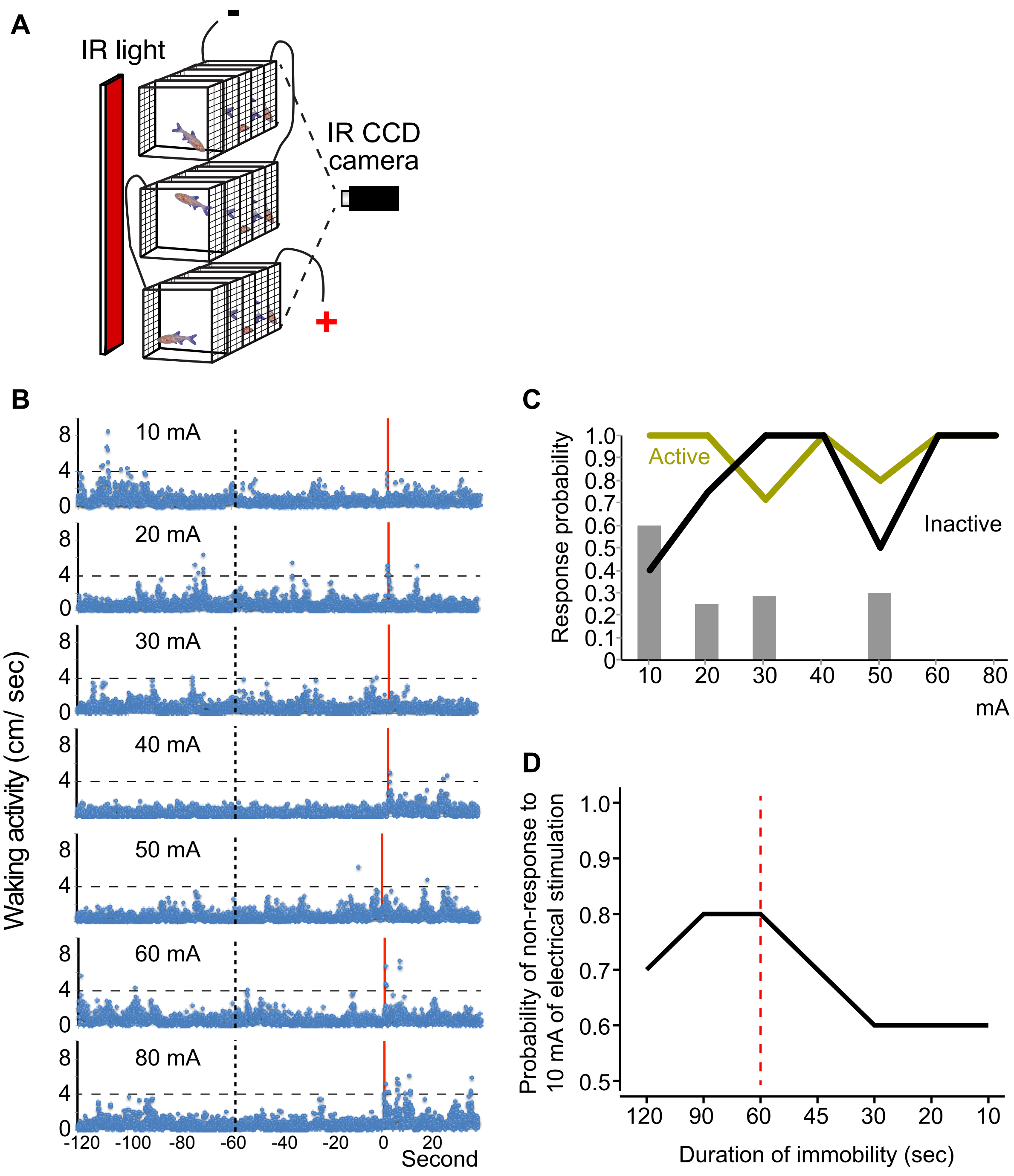

Additional file 1. Parameter selection for sleep-like state in *A. mexicanus*.
(A) Experimental set up for the electrical stimulation. Each 8.7 L recording chamber housing 5 individual fish was fitted with stainless steel-mesh sidewall. Each chamber was connected sequentially and was applied the electrical stimuli in the range of 10 – 80 mA. Fish were illuminated by an array of infrared (IR) light and recorded by IR CCD camera. The behavioral response was measured from 0.5 sec to 30 seconds following electrical stimulation. In addition, the video frame ratio is 15 frames/sec, so that frames were captured every 66.7 msec. (B) Examples of swimming activity for a surface fish. Horizontal represent threshold level of locomotor activity. Red lines indicate the point when we gave electrical stimulation using indicated amperes. Black vertical dotted lines indicate the time a minute before the stimulation and the fish activity between a minute before the stimulation and the stimulation time is a good predictor of the fish response after the stimulation, that is, if fish is active more than threshold level (4 cm/ sec) in this time frame, fish have higher probability to respond to the electrical stimulus. (See D too). (C) The fish response probability in active surface fish (more than 4 cm/sec activity in a 1 min-time frame before the stimulation, shown by black horizontal dotted line) and in inactive surface fish (less than 4 cm/sec activity in a 1 min-time frame). The response probability to 10 mA stimulus is reduced in inactive fish, suggesting increased response threshold. Grey bars indicate the difference of response probability between active and inactive fish. (D) Probability of non-response to the 10 mA electrical stimulation. Fish showed higher response threshold if fish were still for 60 - 90 sec. One minute immobility, therefore, is taken as a sleep-like state in this study (red dotted line).
