# Supplementary material for: Distinct genetic architecture underlies the emergence of sleep loss and prey-seeking behavior in the Mexican cavefish
Source: BMC Biol. 2015 Feb 20;13:15. doi: 10.1186/s12915-015-0119-3 (PMC4364459; doi:10.1186/s12915-015-0119-3)
Supplement: Additional file 2: — Diurnal activity patterns in surface fish and Pachón cavefish. [file 12915_2015_119_MOESM2_ESM.pdf]

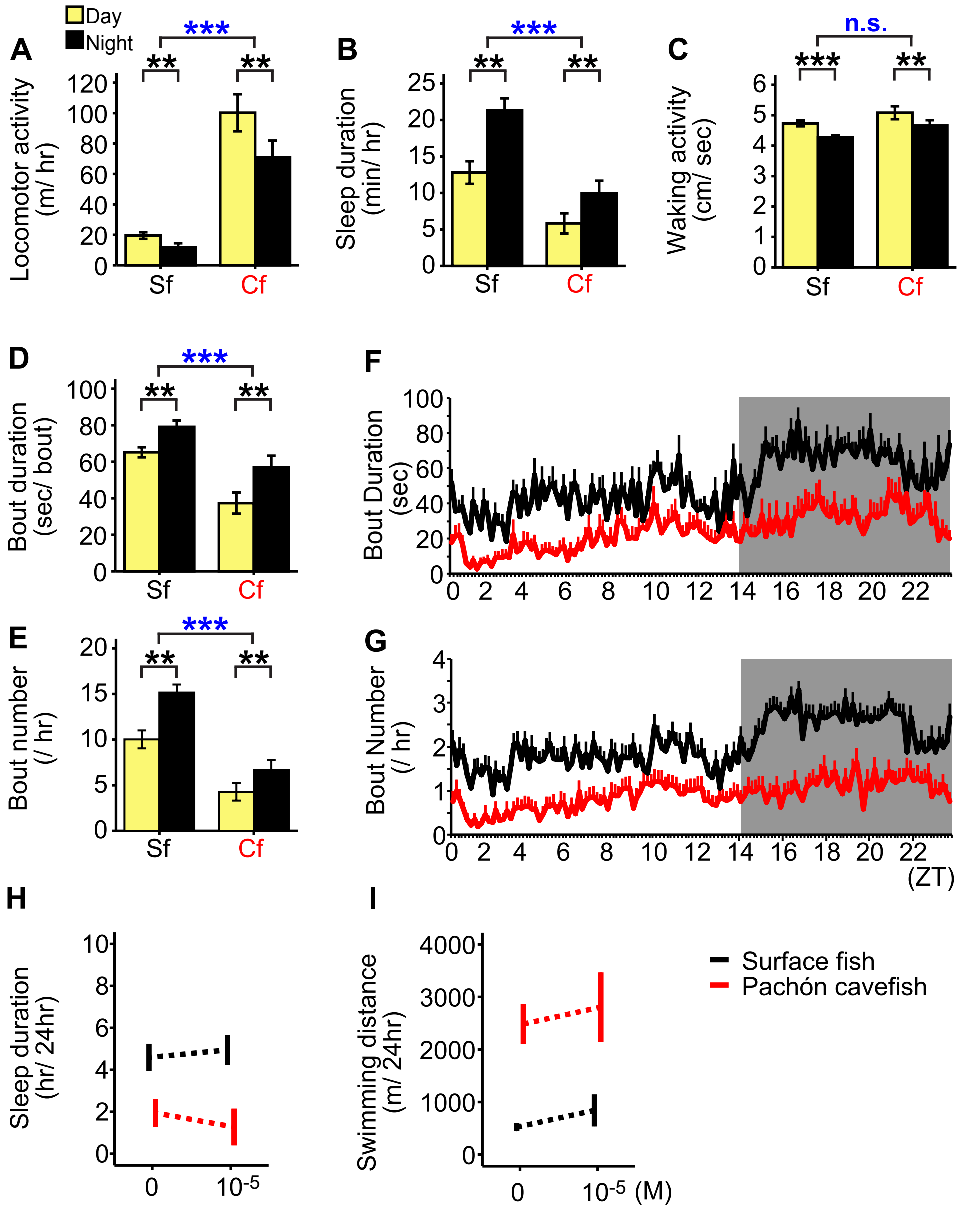

Additional file 2. Diurnal activity patterns in surface fish and Pachón cavefish.
(A-F) Day (yellow bars) and night (black bars) scores of locomotor activity (m/ hr, A), sleep duration (min/ hr, B), waking activity (cm/ sec, C), bout duration (sec/ bout, D) and bout number (/hr, E). Blue stars or non-significant (n.s.) are for the comparison between surface fish (Sf) and Pachón cavefish (Cf). Cavefish and surface fish changed their day-night activities significantly. Two-way ANOVA statistics for each phenotype are: for locomotor activity (A) between surface fish (Sf) and cavefish (Cf): $F_{1,83} = 35.1$, $P < 0.001$, between day and night: $F_{1,83} = 53.3$, $P < 0.001$, interaction between population and day-night: $F_{1,83} = 18.4$, $P < 0.001$; for sleep duration (B) between Sf and Cf: $F_{1,83} = 19.8$, $P < 0.001$, between day and night: $F_{1,83} = 40.9$, $P < 0.001$, interaction between population and day-night: $F_{1,83} = 6.0$, $P = 0.016$; for waking activity (C) between Sf and Cf: $F_{1,83} = 3.3$, $P = 0.074$, between day and night: $F_{1,83} = 40.9$, $P < 0.001$, interaction between population and day-night: $F_{1,83} = 0.0$, $P = 0.838$; for bout duration (D) between Sf and Cf: $F_{1,83} = 16.3$, $P < 0.001$, between day and night: $F_{1,83} = 30.1$, $P < 0.001$, interaction between population and day-night: $F_{1,83} = 0.9$, $P = 0.356$; for bout number (E) between Sf and Cf: $F_{1,83} = 31.0$, $P < 0.001$, between day and night: $F_{1,83} = 48.6$, $P < 0.001$, interaction between population and day-night: $F_{1,83} = 6.5$, $P = 0.012$. For all analyses, N = 42 and 43 for surface fish and Pachón cavefish, respectively. Difference between day and night activities were tested by post-hoc paired t-test with Bonferroni correction and denoted by black asterisks. *** denotes P < 0.001. ** denotes P < 0.01 (F, G) Average bout duration (sec/ bout) and bout number in every 10 min were measured and plotted in 24 hrs, respectively. Bars at each 10 min represent mean + s.e.m. X-axis represents time (Zeitgeber 24 hrs). Both surface fish (black line) and Pachón cavefish (red line) showed day (ZT 0 – 14) and night (ZT 14 – 24) rhythm. (H, I) A noradrenalin antagonist, Propranolol, did not increase sleep (H) or reduce swimming distance (I) in adult cavefish. N = 10 and 10 for surface fish and Pachón cavefish, respectively. Two-way repeated ANOVA in sleep duration of cavefish: $F_{1.2}$, 10.7 = 0.7, P = 0.443; in swimming distance: $F_{1.5}$, 13.8 = 3.6, P = 0.065. Both F-values are adjusted by Greenhouse-Geisser correction. X-axes indicate the concentration of Propranolol. M: molar.
