# Supplementary material for: Distinct genetic architecture underlies the emergence of sleep loss and prey-seeking behavior in the Mexican cavefish
Source: BMC Biol. 2015 Feb 20;13:15. doi: 10.1186/s12915-015-0119-3 (PMC4364459; doi:10.1186/s12915-015-0119-3)
Supplement: Additional file 3: — Detailed analysis of sleep and activity related traits in multiple cavefish populations. [file 12915_2015_119_MOESM3_ESM.pdf]

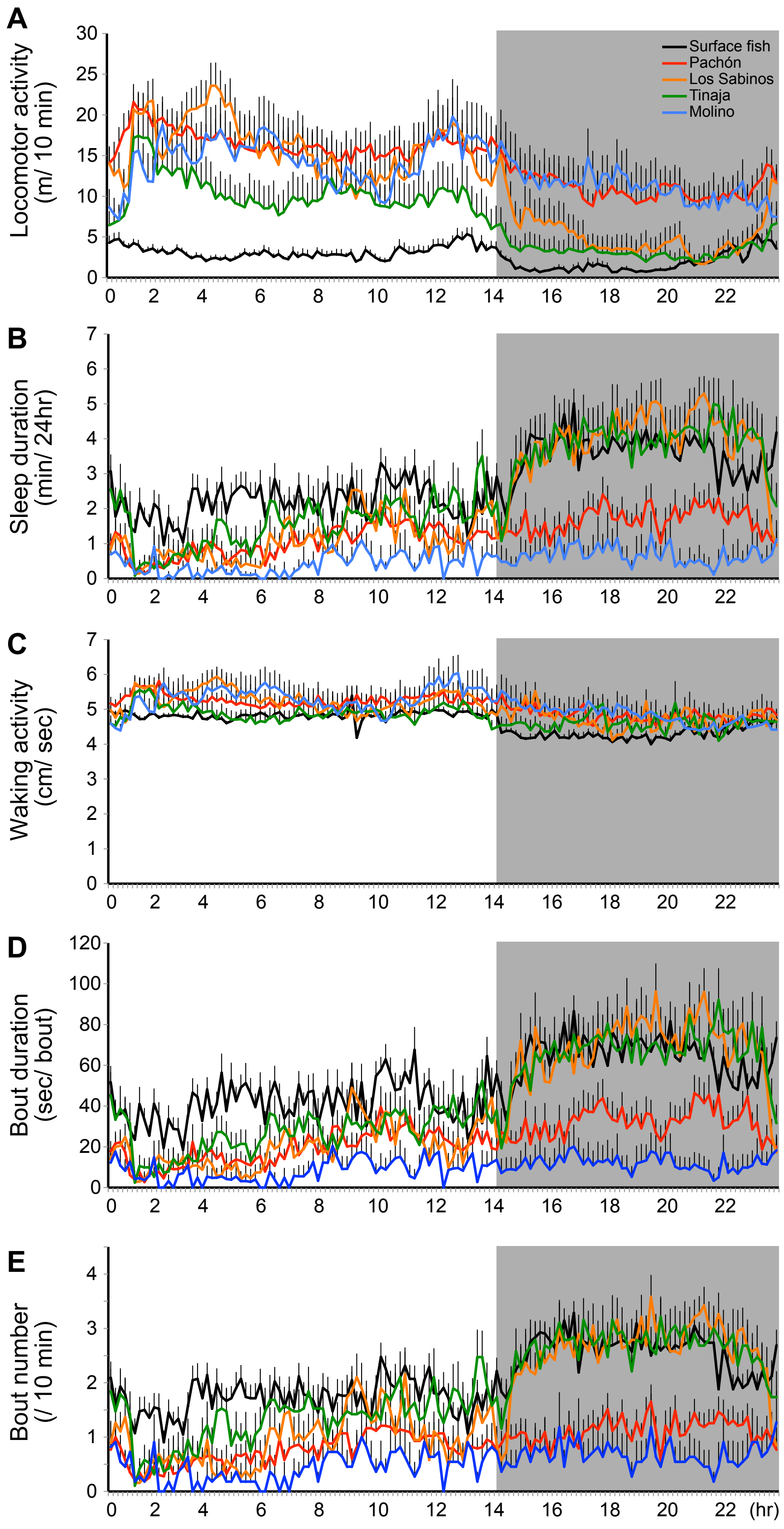

Additional file 3. Detailed analysis of sleep and activity related traits in multiple cavefish populations.

(A) Average locomotor activity (m) per 10 min over 24 hrs observation. (B) Cumulative sleep duration (min) per 10 min over 24 hrs observation. (C) Average waking activity (cm/ sec) in every 10 min in 24 hrs observation. (D) Average bout duration (sec) per 10 min in 24 hrs observation. (E) Cumulative bout number over 24 hrs observation. Bars at each 10 min represent mean + s.e.m. X-axis represents time (Zeitgeber 24 hrs). Black line: surface fish. Red line: Pachón cavefish. Orange line: Los Sabinos cavefish. Green line: Tinaja cavefish. Blue line: Molino cavefish. Pachón and Molino cavefish showed reduced sleep but Los Sabinos and Tinaja cavefish showed comparable diurnal sleep rhythm with surface fish. N = 23, 23, 19, 19 and 11 for surface fish, Pachón, Los Sabinos, Tinaja and Molino cavefish, respectively.
