# Supplementary material for: Distinct genetic architecture underlies the emergence of sleep loss and prey-seeking behavior in the Mexican cavefish
Source: BMC Biol. 2015 Feb 20;13:15. doi: 10.1186/s12915-015-0119-3 (PMC4364459; doi:10.1186/s12915-015-0119-3)
Supplement: Additional file 4: — A summary of the location and effect size of significant QTLs for foraging- and sleep-related traits. [file 12915_2015_119_MOESM4_ESM.pdf]

Linkage Group	Position (cM)	LOD	PVE	Effect size	95% Baysean credible interval (cM)	Zebrafish chromosome (Zv9)
**VAB (N = 227)**			(Total: 17.3)	(0 - 7.68)		
2	23	2.38	4.1	+1.00	20.0 - 90.8	Ch5 (4.7 - 64.6)<br>Ch20 (0.6 - 53.8)<br>Ch24 (12.9 - 34.5)
17	49	5.69	9.6	+1.17	45.0 - 55.0	Ch8 (0.7 - 55.3)
**Locomotor acitivity (N = 58)**			(Total: 43.6)	(8.8 - 4371.0)		
3	42	5.73	32.5	-920.2	39.0 - 44.0	Ch4 (4.1 - 60.2)
22	41	3.42	17.6	+1604.2	37.0 – 44.0	Ch7 (27.8 - 68.4)<br>Ch8 (23.1 - 52.6)

Additional file 4. A summary of the location and effect size of significant QTLs for foraging- and sleep-related traits.

The number of F_2 and F_3 individuals used (N), LOD is the log of odds ratio, and PVE is the percentage of phenotypic variance explained. The summed PVE of all QTL are shown at the top row of each trait (at Total:). Effect size and direction (positive and negative) of QTL refers to the magnitude of shift between homozygotes of surface fish alleles and cavefish alleles. The numbers in parentheses indicate the ranges of phenotypes. The syntenic regions of the zebrafish genome are indicated as chromosome number (syntenic region in mega base pairs, Mbp). Note, PVE at LG 22 QTL for locmotor activity can be inflated because of Beavis effect yet PVE at LG 3 is above detectable level (calculated with qtlDesign; [1, 2])

Linkage Group	Candidate gene	Expression pattern (zfin)	Gene product function
LG 2, 23 cM<br><br>Syntenic to zebrafish ch 5 (4.7 - 64.6 Mb), 20 (0.6 - 53.8 Mb), 24 (12.9 - 34.5 Mb)	eya1 (eyes absent homolog 1)	Placode (nasal, otic, lateral line, trunk muscle), mature lateral line	A transcription factor,possibly maintain developmental plasticity [3]
	cdh2 (N-cadherin2, type 1)	Posterior lateral line primordium, neuromasts, brain, retina, lens, olfactory epithelium, myotome, somite,	Mediating cell adhesion through hemophilic interaction. CDH2 is involved in multiple aspects of development including the lateral line formation [4–6]
	disp1 (Dispatched 1)	Neuroectoderm, oral/pharyngeal ectoderm	Regulating the release of hedgehog protein. Involved in craniofacial formation through organizing the differentiation of neural crest cells [7]
	pcsk5a (proprotein convertase subtilsin/kexin type 5a)	Neuromasts, otic vesicle, central nervous system, liver, intestine.	A serine proteinase facilitating tissue- and substrate-specific processing of protein precursors. Involved in neuromast development and neuromast-dependent behavior [8]
	tbx1 (T-box 1)	Otic vesicle anterior lateral plate mesoderm, heart, pharyngeal arch	A transcription factor, mediating retinoic acid and hedgehog signaling so that controls otholis development [9].
	vgll2a (vestigial-like 2a)	Ventral region of forbrain, notochord, pharyngeal	A transcription co-factor, contributing pharyngeal formation by regulating neural crest cell survival and cartilage development [10].
LG 17, 49 cM<br><br>Syntenic to zebrafish ch 8 (0.7 - 55.3 Mb)	gnat2 (guanine nucleotide binding protein (G protein), alpha transducing activity polypeptide 2)	Eye, retina, pineal gland	A G-protein coupled photoreceptor. null mutation makes the cone insensitive to moderate light intensity [11].
	ptch1 (patched 1)	Retina, ciliary marginal zone, forbrain, hindbrain, hypothalamus, otic vesicle, otic epithelium, gut	Hedgehog negative regulator. Null mutation causes over-proliferation of retina or no phenotype [12].

LG 3, 42 cM<br><br>Syntenic to zebrafish ch 4 (4.1 - 60.2 Mb)	slc6a1l (solute carrier family 6 (neurotransmitter transporter, GABA), member 1, like)	N/A	N/A, GABAnergic system inhibit the activity of arousal system including lateral hypothalamus orexin neuron [13].
LG 22, 41 cM<br><br>Syntenic to zebrafish ch 7 (27.8 - 68.4 Mb), 8 (23.1 - 52.6 Mb), 11 (43.8 - 46.5 Mb)	chrna7 (cholinergic receptor, nicotinic, alpha 7)	Hindbrain, retina	A subunit of Nicotinic acetylcholine receptor mediating sleep – arousal transition, and mutation causes epilepsy [14, 15].
	drd4b (dopamine receptor D4b)	Branchial arch, diencephalon, mid-brain, otic vesicle, spinal cord, telencephalon, tegmentum	Dopamine receptor mediates locomotor activity [16].
	gli2b (GLI family zinc finger 2b)	Cerebellum, diencephalon, dorsal thalamus, hindbrain, optic tectum	A transcription factor contributing cell differentiation expressed higher in oligodendrocyte in the wake state [17].
	tp53i11a (tumor protein p53 inducible protein 11a)	N/A	N/A, tp53 (expressed in brain pancreas, eye, optic tectum, gill, gut) is associated with narcolepsy [18].

Additional file 4 continue

Candidate genes were selected based on their expression pattern, potential contribution to the foraging and neuromast development (VAB QTL), or sleep and/or locomotor activity (Locomotor acitivity QTL). Genes whose mutations impair many tissues (for example, *mcm3*) are excluded. The number of genes after the BioMart filter was 287 for LG2, 73 for LG3, 188 for LG17, and 130 for LG22.

Additional file 4
References:

1. Beavis WD: **QTL Analyses: Power, Precision, and Accuracy**. In *Mol Dissection Complex Trait*. *Volume 3*. Edited by Paterson AH. London: CRC Press; 1998:145–162.
2. Broman KW, Sen S: *A Guide to QTL Mapping with R/qtl*. 1st Editio. New York: Springer-Verlag; 2009:396.
3. Sahly I, Andermann P, Petit C: **The zebrafish *eya1* gene and its expression pattern during embryogenesis**. *Dev Genes Evol* 1999, **209**:399–410.
4. Liu Q, Marrs JA, Azodi E, Kerstetter AE, Babb SG, Hashmi L: **Differential expression of cadherins in the developing and adult zebrafish olfactory system**. *J Comp Neurol* 2004, **478**:269–281.
5. Riley BB, Sweet EM, Heck R, Evans A, McFarland KN, Warga RM, Kane DA: **Characterization of harpy/Rca1/emi1 mutants: Patterning in the absence of cell division**. *Dev Dyn* 2010, **239**:828–843.

6. Kerstetter AE, Azodi E, Marrs JA, Liu Q: **Cadherin-2 function in the cranial ganglia and lateral line system of developing zebrafish.** *Dev Dyn* 2004, **230**:137–43.
7. Schwend T, Ahlgren SC: **Zebrafish con/disp1 reveals multiple spatiotemporal requirements for Hedgehog-signaling in craniofacial development.** *BMC Dev Biol* 2009, **9**:59.
8. Chitramuthu BP, Baranowski DC, Cadieux B, Rousselet E, Seidah NG, Bennett HPJ: **Molecular cloning and embryonic expression of zebrafish PCSK5 co-orthologues: functional assessment during lateral line development.** *Dev Dyn* 2010, **239**:2933–2946.
9. Radosevic M, Robert-Moreno A, Coolen M, Bally-Cuif L, Alsina B: **Her9 represses neurogenic fate downstream of Tbx1 and retinoic acid signaling in the inner ear.** *Development* 2011, **138**:397–408.
10. Johnson CW, Hernandez-Lagunas L, Feng W, Melvin VS, Williams T, Artinger KB: **Vgll2a is required for neural crest cell survival during zebrafish craniofacial development.** *Dev Biol* 2011, **357**:269–81.
11. Brockerhoff SE, Rieke F, Matthews HR, Taylor MR, Kennedy B, Ankoudinova I, Niemi GA, Tucker CL, Xiao M, Cilluffo MC, Fain GL, Hurley JB: **Light Stimulates a Transducin-Independent Increase of Cytoplasmic Ca^{2+} and Suppression of Current in Cones from the Zebrafish Mutant nof**. *J Neurosci* 2003, **23**:470–480.
12. Bibliowicz J, Gross JM: **Ectopic proliferation contributes to retinal dysplasia in the juvenile zebrafish *patched2* mutant eye.** *Invest Ophthalmol Vis Sci* 2011, **52**:8868–77.
13. Rihel J, Schier AF: **Sites of action of sleep and wake drugs: insights from model organisms.** *Curr Opin Neurobiol* 2013, **23**:831–40.
14. Marini C, Guerrini R: **The role of the nicotinic acetylcholine receptors in sleep-related epilepsy.** *Biochem Pharmacol* 2007, **74**:1308–14.
15. Zirger JM, Beattie CE, McKay DB, Thomas Boyd R: **Cloning and expression of zebrafish neuronal nicotinic acetylcholine receptors**. *Gene Expr Patterns* 2003, **3**:747–754.
16. Boehmler W, Carr T, Thisse C, Thisse B, Canfield V a, Levenson R: **D4 Dopamine receptor genes of zebrafish and effects of the antipsychotic clozapine on larval swimming behaviour.** *Genes, brain Behav* 2007, **6**:155–166.
17. Bellesi M, Pfister-Genskow M, Maret S, Keles S, Tononi G, Cirelli C: **Effects of sleep and wake on oligodendrocytes and their precursors.** *J Neurosci* 2013, **33**:14288–300.
18. Wieczorek S, Jagiello P, Arning L: **Screening for candidate gene regions in narcolepsy using a microsatellite based approach and pooled DNA**. *J Mol Med* 2004, **82**:696–705.
