# Supplementary material for: Distinct genetic architecture underlies the emergence of sleep loss and prey-seeking behavior in the Mexican cavefish
Source: BMC Biol. 2015 Feb 20;13:15. doi: 10.1186/s12915-015-0119-3 (PMC4364459; doi:10.1186/s12915-015-0119-3)
Supplement: Additional file 5: — The dopamine receptor antagonist haloperidol modulates sleep and locomotor activity in A. mexicanus. [file 12915_2015_119_MOESM5_ESM.pdf]

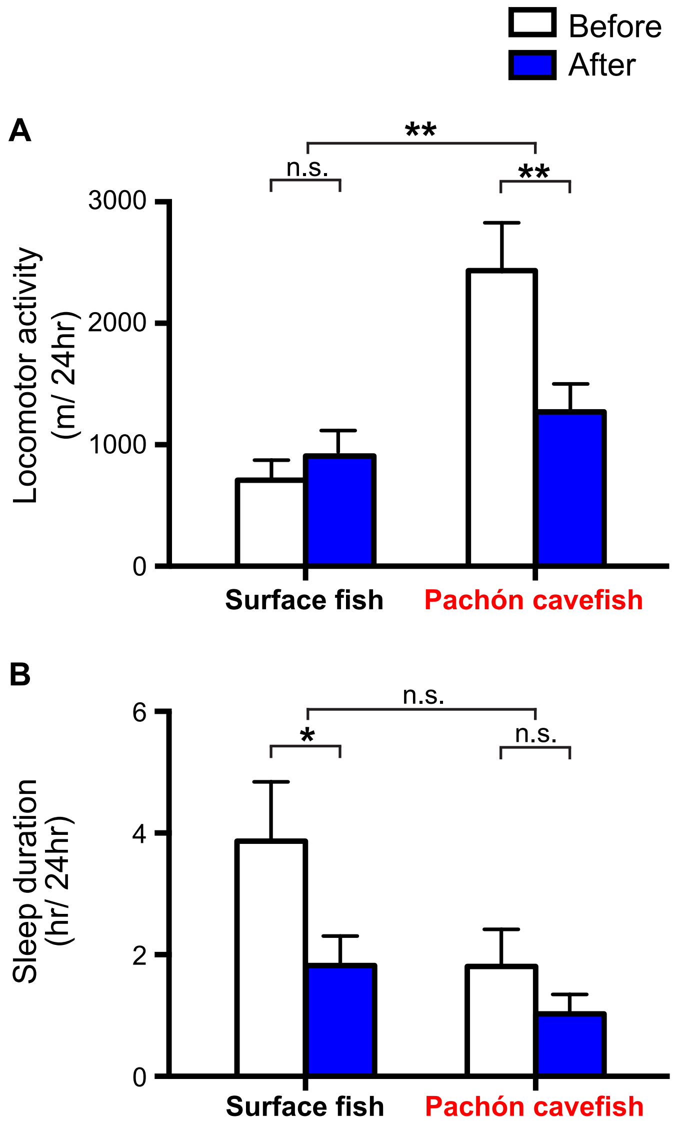

Additional File 5. The dopamine receptor antagonist haloperidol modulates sleep and locomotor activity in *A. mexicanus*.

Following 24hrs of baseline recordings (control) subjects were treated with 10 μM haloperidol over 24hrs to measure behavior modification [1, 2]. (A) Significant reduction of locomotor activity was detected between before and after treatment of haloperidol (repeated measures two-way ANOVA: $F_{1,28} = 9.1$, $P = 0.005$) and between surface fish and Pachón cavefish ($F_{1,28} = 9.7$, $P = 0.004$). Also, there is significant interaction between treatment and population ($F_{1,28} = 17.5$, $P < 0.001$). In detail, surface fish did not show the significant change in locomotor activity after the treatment ($t_{14} = -1.3$, $P = 0.462$) but Pachón cavefish did ($t_{14} = 4.1$, $P = 0.002$). (B) A significant reduction of sleep duration was observed after the haloperidol treatment (repeated-measures two-way ANOVA: $F_{1,28} = 8.2$, $P = 0.008$) while the differences were under detection level between surface fish and Pachón cavefish ($F_{1,28} = 3.5$, $P = 0.073$) and in the interaction between treatment and population ($F_{1,28} = 1.7$, $P = 0.202$). In the posthoc analysis, the total hours of sleep was significantly decreased in surface fish ($t_{14} = 2.8$, $P = 0.030$) but not in Pachón cavefish ($t_{14} = 1.4$, $P = 0.510$).

Note, postdoc tests were performed with Bonferroni correction to account for multiple comparisons. N = 15 and 15 for surface fish and Pachón cavefish, respectively. ** denotes $P < 0.01$. ** denotes $P < 0.05$. n.s.: not significant .
